# Pediatric Cancer as a Factor of Changes in the Family

**DOI:** 10.3390/ijerph19095002

**Published:** 2022-04-20

**Authors:** Aleksandra Dąbrowska, Iwona Malicka

**Affiliations:** Department of Physiotherapy, Wroclaw University of Health and Sport Sciences, 51-612 Wroclaw, Poland; adabrowska3a1@gmail.com

**Keywords:** pediatric cancer, resilience, parents, patient care

## Abstract

The occurrence of pediatric cancer is an example of a non-normative situation that reorganizes family life. The aim of the study was to evaluate the functioning of a family with a child affected by cancer. The study was conducted on 339 families. The study group consisted of 153 families with children with cancer (mean age 36.4 ± 6.8 years). The control group was composed of 186 families with healthy children (mean age 39.0 ± 6.3 years). All of them completed the author’s survey questionnaire on family functioning and the Resilience Measurement Scale (RMS). A statistically significant association was found between the place of residence (*p* < 0.001), education (*p* < 0.001), assessment of the material status (*p* < 0.001) and employment structure (*p* < 0.001) of parents and the membership in the study group or the control group. Statistical significance was achieved for the main effects as measured on the RMS (5 factors and 2 groups). The study group showed consistently lower levels of the factors of the scale. Statistically significant observations were reported for Factor 1 (perseverance, proactive approach) and Factor 4 (tolerance to failure, life as a challenge) (12.0 vs. 14.5, *p* < 0.001, 13.4 vs. 14.2, *p* = 0.04, respectively). Parents of children with cancer were characterized by lower persistence, determination and tolerance to failure, which could affect the quality of life of the whole family. In addition, different socio-economic conditions of family functioning were found in families with children with cancer as compared to families with healthy children.

## 1. Introduction

Next to accidents, injuries and intoxication, cancer is the second most prevalent cause of death in children and adolescents aged 1–19 years in the European Union and in Poland [1].

According to the International Classification of Childhood Cancer (ICCC), the most common pediatric cancers include leukemias, lymphomas and tumors of the central nervous system [2,3].

Of note, cancer disease does not only affect the patient, and its impact must be considered in a broader social context. All diseases, especially chronic conditions, are a specific part of the family structure. They become a part of life for its members, often changing it irreversibly [4].

The occurrence of pediatric cancer is an example of a non-normative situation that reorganizes family life. Parents must begin to perform new functions and undertake new tasks to cope with the new demands [5,6].

Many functional changes occur in the family due to pediatric cancer. All family members experience enormous stress and emotional strain, which affects relationships inside and outside the family as well as the family’s life plans. It is necessary to maintain balance by finding one’s own ways of adaptation to survive this traumatic situation. Functioning of the family is changed and all the aspects are affected, including financial, educational, and emotional issues. Parents very often struggle with overload and the excess of responsibilities, which results in lack of time for rest and causes disturbances related to performing professional duties or even resignation from work. Feelings of rejection are often reported in other family members (mostly in healthy siblings). Tension and disagreement may also occur between spouses. They are related to a sense of mutual isolation because one parent takes care of the child and therefore does not work professionally, while the other parent works as much as possible to support the family [7,8,9,10].

There are also reports that indicate the presence of subclinical posttraumatic stress symptoms (PTSS) in affected children and their parents. This term describes a normative response to a crisis situation rather than a form of the disorder that meets the diagnostic criteria for posttraumatic stress disorder (PTSD) [11,12].

The literature review did not show a clear presence or absence of differences in the functioning of families with children with cancer compared to families of healthy children. Long and Marsland [8] in their systematic review indicated that the functioning of families with children with cancer was not significantly different from families with healthy children. However, Hosoda [13] showed such differences.

Therefore, the aim of the study was to assess the functioning of families with children affected by cancer. 

The primary aim was to assess the resilience of parents of children with cancer in relation to parents of healthy children. The secondary aim was focused on analyzing the socio-economic situation of the families studied.

## 2. Materials and Methods

### 2.1. Study Group 

The study was conducted on 339 families. The study group consisted of 153 families with children with cancer. The survey questionnaire was completed by 141 females and 12 males (mean age 36.4 ± 6.8 years). The control group included 186 families with healthy children. The female-to-male ratio was 157:29 (mean age 39.0 ± 6.3 years). In the study group, the inclusion criteria were the age of children (<18 years) and the diagnosis of cancer. In the control group, the inclusion criteria were as follows: age of children (<18 years) and the absence of any chronic, genetic or cancer disease. Their parents were informed about the aim of the study and the possibility to withdraw at any stage. The study was conducted in the form of a survey questionnaire without any intervention or experimental structure. The participants gave their informed consent, and the study was conducted in accordance with the Helsinki Declaration and under the ethical and legal supervision of the Department of Physiotherapy of the Wroclaw University of Health and Sport Sciences. The study was conducted as part of the project known as “Evaluation of the psychophysical state of people treated for cancer” (consent no 7/2018). In the case of children, the study involved whole families. The manuscript showed the results of parents of children with cancer.

### 2.2. Research Methods 

The data were obtained from the Supraregional Center of Pediatric Oncology Centre in Wroclaw and Internet forums for parents of children with cancer and parents in general. 

The data were collected electronically, the questionnaire was made available through a link. The request to complete the questionnaire was addressed to one parent only. In the case of children with cancer, this was the parent who cared for the child in hospital during treatment. In the case of healthy children, it was the parent who spent most of the time with the child.

The participants completed the author’s survey questionnaire concerning family functioning and the Resilience Measurement Scale (RMS). 

Before completing the questionnaire, the participant had read information about the aim and the procedure of the study and consented to the study by marking the appropriate answers in the form.

The survey questionnaire included questions about age, gender, the place of residence, education and the financial situation of parents. Additionally, it included the questions about diagnosis, age at cancer diagnosis in the child and the manner of detecting the first symptoms. Other questions were also related to changes in the family situation (parental separation, necessity to leave work), care of the child in the hospital and conversations with the child about death and dying.

The Resilience Measurement Scale developed by Ogińska-Bulik and Juczyński (2008) consists of 25 items concerning (1) perseverance and proactive approach, e.g., “I make efforts to cope no matter how difficult the problem is”, (2) openness to new experiences, sense of humor, e.g., “Even in a difficult situation I find something to laugh about”, “I can look at a situation from different points of view”, (3) personal coping skills and tolerance of negative emotions, e.g., “I concentrate and think clearly in stressful situations”, “I consider myself a strong person”, (4) tolerance to failure, treating life as a challenge, e.g., “I adapt easily to new situations”, “I can learn from past failures”, (5) optimistic attitude to life and the ability to organize oneself in difficult situations, e.g., “Experiencing difficulties motivates me to act”. The above subscales can be assessed as separate factors or can be related to the whole scale by summing up and/or extracting the sten scores. A higher resilience level is indicated by a higher number of points. The answers were given using a 5-item Likert scale (from 0—*strongly disagree*, to 4—*strongly agree*) [14].

The scale reliability measured by Cronbach’s alpha was 0.89 for the whole scale. The reliability of the five subscales was similar and ranged from 0.67 to 0.75 (0.72, 0.68, 0.74, 0.67, 0.75 for Factors 1, 2, 3, 4 and 5, respectively). Since each subscale consisted of 5 items, the obtained results were considered satisfactory [14].

### 2.3. Statistical Analysis 

Descriptive statistics, Chi-square test (for socio-economic characteristics) and Student’s *t*-test (for the difference between genders in the study group and for the difference between the RMS in the groups) were used in the statistical analysis. Additionally, the analysis of variance with the Bonferroni test was applied for the RMS factors. The normality of distribution was verified using the Shapiro–Wilk test and the homogeneity of variance with the Levene’s test. A significance level of *p* = 0.05 was established. The calculations were performed using Statistica 12 StatSoft Poland (StatSoft, Inc., Tulsa, OK, USA).

## 3. Results

**Figure 1:**
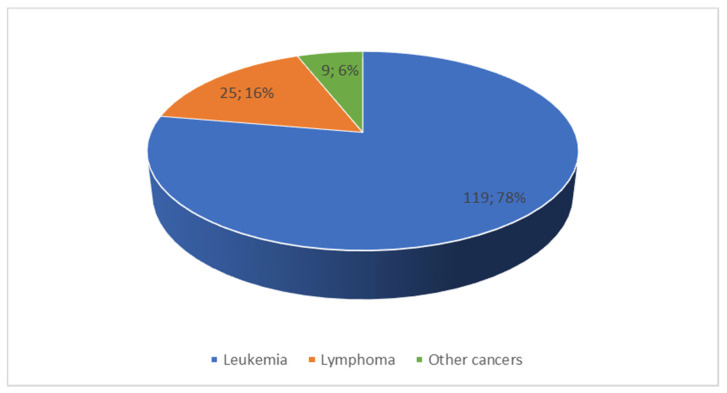
Diagnoses in children with cancer.

In the study group, the mean age at diagnosis was 5.76 years (±4.54) with no significant difference between genders (*t* = −0.30, *p* = 0.75). Most children were diagnosed with leukemia (78%) and lymphoma (16%); (Figure 1). 

**Figure 2:**
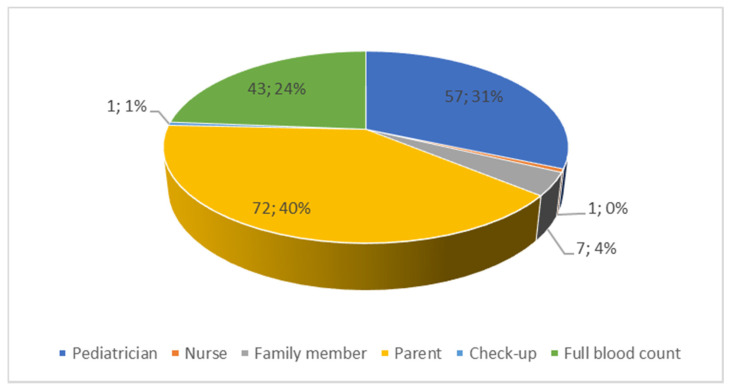
Recognition of the first symptoms in the child.

The first symptoms of cancer disease were mostly observed by parents (40%), pediatricians (31%) or were detected at the follow-up examination (full blood count) (24%). The detailed data are shown in Figure 2.

**Figure 3:**
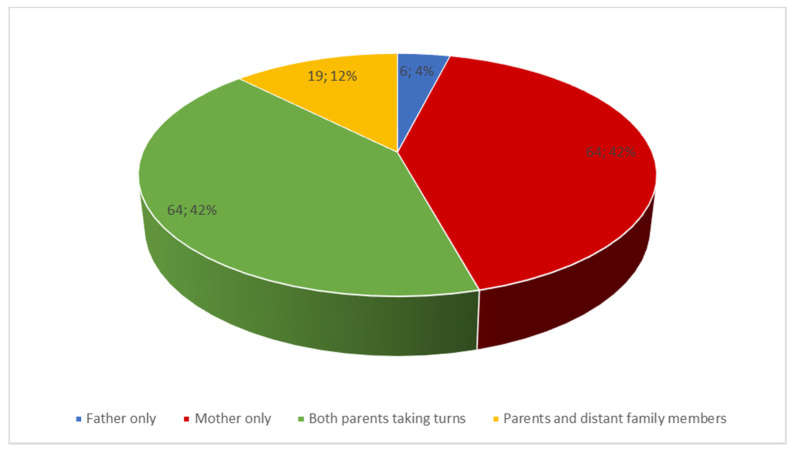
Care of the child during hospitalization.

During the hospital stay of the child, care was mostly provided by the mother (42%) or both parents who took turns (42%; Figure 3).

**Figure 4:**
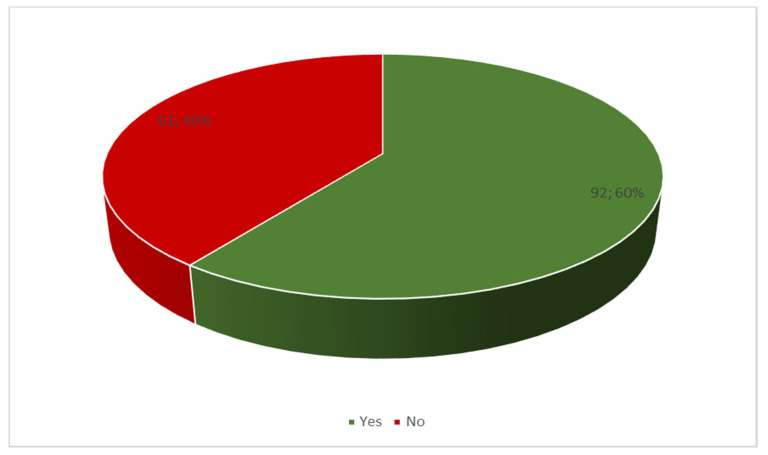
Distribution of responses to the question about crying in the presence of the child due to the child’s deteriorating health.

In the study group, most parents (60%) reacted with crying in the presence of their child because of their child’s deteriorating health (Figure 4).

**Figure 5:**
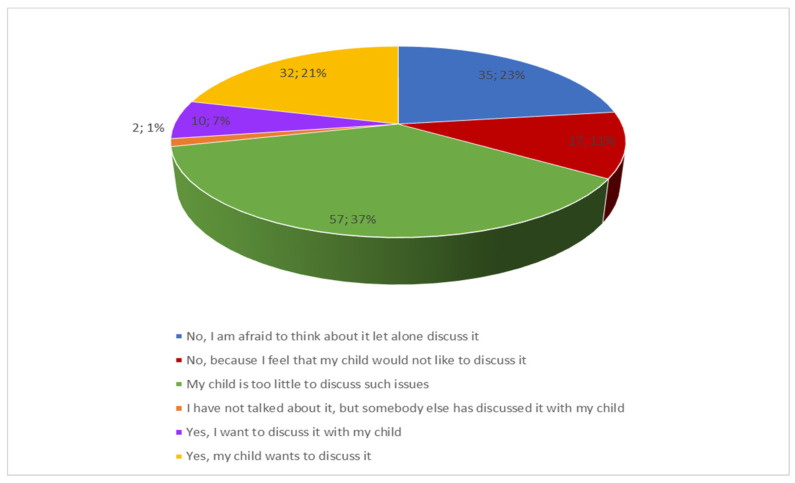
Distribution of responses to the question about conversations with the child about death and dying.

When asked about conversations with their child about death and dying, most participants reported that their child was too little to discuss such issues (37%). Other parents/caregivers indicated affirmative (29%) or negative responses (34%). The detailed distribution of responses is given in Figure 5. 

There was a statistically significant relationship between the place of residence (*p* < 0.001), education (*p* < 0.001), the financial status (*p* < 0.001) and employment structure of parents (*p* < 0.001) and the membership in the study group or the control group (Table 1). However, no differences were reported in terms of upbringing of the child (*p* = 0.2; Table 1). Children were mostly raised in complete families in both the study group and controls (94.1% vs. 90.9%, respectively).

Statistical significance was achieved for the main effects as measured on the RMS (five factors and two groups). The results of the analysis of variance are given in Table 2.

**Figure 6:**
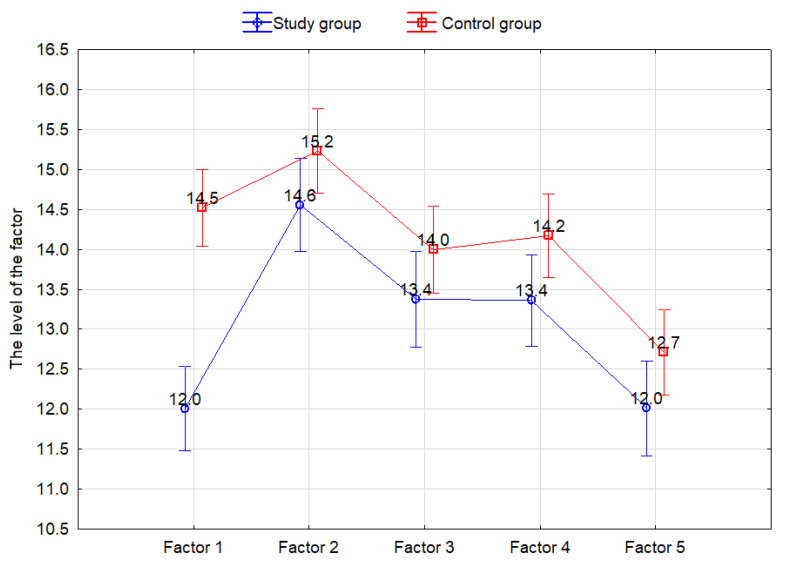
Distribution of the factors of the Resilience Measurement Scale depending on the group: Factor (1) perseverance and proactive approach, Factor (2) openness to new experiences, sense of humor, Factor (3) personal coping skills and tolerance to negative emotions, Factor (4) tolerance to failure, treating life as a challenge, Factor (5) optimistic attitude to life and the ability to organize oneself in difficult situations.

It was found that Factor 5 (optimistic attitude to life and the ability to organize oneself in difficult situations; 12.0 in the study group vs. 12.7 in the controls; M = 12.35) showed the lowest level, and it was statistically significant in relation to other factors (*p* < 0.001). On the other hand, Factor 2 (openness to new experiences and sense of humor; 14.6 in the study group vs. 15.2 in the controls; M = 14.9) showed the highest level and was significantly higher than other factors (*p* < 0.001). The detailed data are given in Figure 6 and Table 3.

In addition, the study group showed consistently lower levels of the factors in the RMS. This finding was statistically significant for Factor 1 (perseverance, proactive approach) and Factor 4 (tolerance to failure, treating life as a challenge) (12.0 vs. 14.5, *p* < 0.001; 13.4 vs. 14.2, *p* = 0.04, respectively). The detailed data for the study groups and the RMS are given in Figure 6 and Table 4.

**Figure 7:**
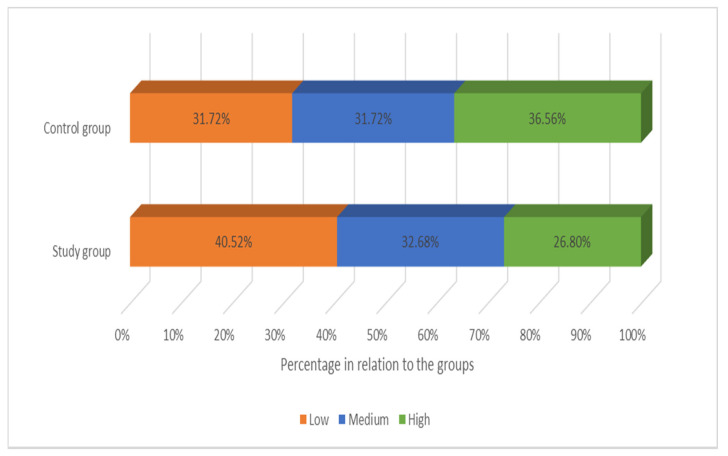
Severity of resilience depending on the group.

However, no statistical significance was found in the differences between the groups when all factors of the scale were added (*t* = −1.43, *p* = 0.15) or when given in the form of stens (chi^2^ = 4.36, *p* = 0.12). The sten data on the severity of resilience are given in Figure 7. 

## 4. Discussion

A serious illness of the child, including cancer, is highly stressful for parents and/or caregivers and may significantly affect the support for the child, thus influencing their well-being and quality of life. It is therefore important to identify resilience resources of parents and/or caregivers, which should translate into adequate support for these people and should improve the functioning of the whole family. 

The study attempted to assess resilience, which is defined in many ways. One of the definitions describes it as a mechanism of positive adaptation to changing life conditions that protects against psychological stress [14,15]. Resilience is therefore associated with the following personality constructs: emotional stability, openness to experience, optimism, a sense of coherence (especially a sense of meaningfulness), a sense of control or self-efficacy. Experiencing positive emotions is an inherent element of resilience [14]. It can be regarded as an outcome of interactions, or can be analyzed through factors.

When resilience was assessed in the distribution into particular factors, lower scores were found in parents of children with cancer. A significantly lower finding was related to Factor 1 (perseverance, proactive approach, e.g., “I make efforts to cope no matter how difficult the problem is”) and Factor 4 (tolerance to failure, treating life as a challenge, e.g., “I adapt easily to new situations”, “I can learn from past failures”). 

The diagnosis of cancer in a child and the beginning of aggressive cancer treatment affect the whole family. It is a difficult and stressful situation for both the child and their parents/caregivers. Additionally, a good prognosis and return to the status before the disease are not always possible. Therefore, all family members should be determined in their actions and should also accept treatment-related adverse effects. Significantly lower scores on Factors 1 and 4 based on the RMS may indicate fatigue and frustration resulting from long-term medical procedures characterized by heavy psychophysical burden of the child and their parents/caregivers. Emotional and physical exhaustion of parents of children with cancer disease was confirmed by Luo et al. [16]. Additionally, in their meta-analysis involving 58 studies, van Warmerdam et al. identified co-existing problems. Compared to parents of healthy children, parents of children with cancer were significantly more likely to experience anxiety (21%), depression (28%) and PTSD (26%) [17]. These conditions may also be associated with the feeling of resilience, as they translate into a decreased ability to cope with new stressful situations such as the child’s illness and the long-term effects of treatment and rehabilitation. In the present study, most parents reacted by crying in the presence of their child. 

Lower resilience in the group of parents of child cancer survivors was also shown by Eilertsen et al. [18] (compared to parents of healthy children), Rosenberg et al. [12] (compared to population norms), and Ye et al. [19] (regarding Chinese society). Luo et al. [16] found that the level of resilience of parents of children with cancer was additionally associated with their quality of life. Individuals with a high level of resilience showed an optimal level of mobilization adapted to the undertaken activities, thus saving the resources involved [20]. As a result, they achieved a better quality of life.

In addition, pediatric cancer can become a threatening factor to the integrity of the family and can disrupt its development. The experience of a child’s cancer is a highly disturbing and chronic life event that goes beyond the survivor and affects the entire family system [21]. 

Our study indicated significant socio-economic relationships in families functioning with a child with cancer. The relationship was found between parental education and the risk of cancer disease in their children. A similar relationship was shown in the adult population by Mouw et al. In their study, lower education correlated with higher cancer incidence in men and women in the United States [22].

Residence in rural areas and small towns was associated with a higher cancer risk, as reported by Oliver et al. [23]. Moreover, many studies indicated that the place of residence was related to cancer survival rates, which were lower among the residents of rural areas [24,25].

Additionally, our study showed that one parent had to leave work to care for the child, which is in line with the findings of other studies [26,27,28]. 

Most children with cancer are raised in complete families, which is a beneficial phenomenon, especially in a situation of reduced income due to reduced employment. These findings were also confirmed by Baruch [29], who also stressed the possibility of strengthening family relationships caused by a crisis related to pediatric cancer. Strengthening the relationship between spouses and the child as part of the adjustment to life after the diagnosis of cancer was also demonstrated by Brody and Simmons [30].

After considering the above data, it seems crucial to develop therapeutic interventions that could increase resilience of parents of children with cancers thus improving their quality of life. The need for support was also stressed by other authors [16,31,32,33]. 

### Limitations

This study had several important limitations.

(1) The survey questionnaires were completed only by volunteers from one region of the country (Lower Silesia, Poland). Therefore, the results cannot be extrapolated to the whole population.
(2) The survey questionnaires were completed electronically without the assistance of health care professionals. As a result, neither the diagnosis nor the current health status of children was verified, which may have affected the level of resilience.
(3) Only one parent per family was enrolled in the study. We did not evaluate dyadic or intrafamily resilience. We did not include data on parent–child or parent-to-parent relationships, which limits conclusions for the whole family.
(4) We did not include an analysis of resilience in relation to socio-economic factors. We did not consider time elapsed since the diagnosis or treatment time in the study group. We lacked power and sample diversity to conduct relevant subgroup analyses.
(5) We did not include information on whether the parents received psychological support or other psychotherapeutic interventions that could have influenced the results.

The above data should be considered in future studies.

## 5. Conclusions

Parents of children with cancer were characterized by reduced persistence, lower proactive approach and tolerance to failure, which in turn could affect the quality of life of the whole family. In addition, different socio-economic conditions were observed in families with children affected by cancer compared to families with healthy children.

## Figures and Tables

**Table 1:** Socio-economic characteristics of the study group and controls.

Feature	Total (N = 339)	Study Group (N = 153)	Control Group (N = 186)	Chi-Squared Test	Study Group vs. Control Group *p*
**Place of residence**					<0.001 *
Village	73	54	19		
Town (<50,000)	75	26	49		
City (50,000–100,000)	34	25	9	60.2	
Large city (>100,000)	157	48	109		
**Education**					<0.001 *
Primary/vocational	17	14	3		
Secondary	68	43	25		
Higher (Bachelor’s degree)	48	24	24	28.28	
Higher (Master’s degree)	206	72	134		
**Financial situation**					<0.001 *
Satisfactory	300	145	155		
Unsatisfactory	32	2	30	227.64	
Poor	7	6	1		
**Employment structure**					<0.001 *
The parent does not work	108	96	12		
One spouse does not work	28	18	10	156.24	
Both parents work	203	39	164		
**Upbringing of the child**					0.2
by both parents	313	144	169	3.21	
by one parent	19	8	11		
with a partner who is not the child’s parent	7	1	6		

* statistical significance (*p* < 0.05).

**Table 2:** Analysis of variance for Resilience Measurement Scale (RMS).

Effect	*F*	*p*
Group	8.867	0.003 *
FACTOR	92.699	<0.001 *
FACTOR × Group	18.344	<0.001 *

* statistical significance (*p* < 0.05).

**Table 3:** The level of statistical significance for the factor of the Resilience Measurement Scale (Bonferroni test).

FACTOR	1 13.25	2 14.9	3 13.7	4 13.8	5 12.35
1		<0.001 *	0.13	0.02 *	<0.001 *
2	<0.001 *		<0.001 *	<0.001 *	<0.001 *
3	0.13	<0.001 *		1.00	<0.001 *
4	0.02 *	<0.001 *	1.00		<0.001 *
5	<0.001 *	<0.001 *	<0.001 *	<0.001 *	

* statistical significance (*p* < 0.05).

**Table 4:** The level of statistical significance between the groups for the factors of the Resilience Measurement Scale (Bonferroni test).

FACTOR	1	2	3	4	5
Study group vs. control group	<0.001 *	0.09	0.13	0.04 *	0.08

* statistical significance (*p* < 0.05).

## Data Availability

Data available upon request.
